# Obituary

**Published:** 1915-10-23

**Authors:** 


### Obituary.

We regret to record the death of the Rev. C. E. Doudney, vicar of St. Luke's Church, Bath, which occurred on Saturday last, October 16, as the result of abdominal shrapnel wounds received as he was on his way to bury a soldier at the Front. It will be remembered by our readers that some months back mention was made in these columns of the valuable account to which Mr. Doudney turned his hobby of electricity when he was sent as a chaplain to a hospital in France. There was a need of an *x*-ray operator, and Mr. Doudney, who was well known as an expert amateur electrician, stepped into the gap and combined the twofold duties of ministering to the body and soul. There is a particular sadness in his death, for he was to have returned from France to be present the following Monday (St. Luke's Day) at the patronal festival of his church in Bath.

